# Complications Following Lingual Tonsillectomy: Analysis of the National Surgical Quality Improvement Program (NSQIP) Database 

**DOI:** 10.7759/cureus.98169

**Published:** 2025-11-30

**Authors:** Beatrice R Bacon, Shiven Sharma, Francesca Viola, Gaayathri Varavenkataraman, Michele M Carr

**Affiliations:** 1 Otolaryngology - Head and Neck Surgery, University of Pittsburgh Medical Center, Pittsburgh, USA; 2 Otolaryngology, Icahn School of Medicine at Mount Sinai, New York, USA; 3 Otolaryngology, Jacobs School of Medicine and Biomedical Sciences at the University at Buffalo, Buffalo, USA; 4 Otolaryngology, Jacobs School of Medicine and Biomedical Sciences, University at Buffalo, Buffalo, USA

**Keywords:** acs-nsqip, hemorrhage, lingual tonsillectomy, morbidity, mortality, surgical complications

## Abstract

Objective

The objective of this study was to describe the postoperative morbidity of adults undergoing lingual tonsillectomy.

Methods

This is a retrospective study performed at an academic medical center. Adults who underwent lingual tonsillectomy were identified using the 2014-2022 American College of Surgeons National Surgical Quality Improvement Program (ACS-NSQIP) database via Current Procedural Terminology (CPT®) code 42870. Data collected included patient demographics, comorbidities, length of stay, readmissions, reoperations, and postoperative complications. Linear regression was used to analyze variable effects on length of stay.

Results

Two hundred and thirty-two patients were included, 129 (55.6%) females and 103 (44.4%) males, with a mean age of 51.6 years (range 18-87 years). Thirty (12.9%) were diabetic, 48 (20.7%) were smokers, and 76 (32.8%) had hypertension treated with medications. Length of stay ranged from 0 to 19 days, with a median of one day, and diabetes was associated with increased length of stay (p=0.004). Within 30 days post-operation, there was one (0.4%) mortality. Complications included three (1.3%) cases of pneumonia, three (1.3%) cases of urinary tract infections, two (0.9%) reintubations, two (0.9%) cases requiring mechanical ventilation for more than 48 hours, one (0.4%) case of septic shock, and one (0.4%) case requiring cardiopulmonary resuscitation. Three (1.3%) patients returned to the operating room, and three (1.3%) were readmitted for a related reason. There were two cases of postoperative hemorrhage within 30 days.

Conclusion

This analysis demonstrated a low risk for morbidity and mortality among lingual tonsillectomy patients.

## Introduction

Lingual tonsillectomy (LT) is a rarely performed procedure when compared to palatine tonsillectomy or adenoidectomy. However, it has been performed at an increased frequency in recent years, perhaps due to innovations in surgical techniques or increased knowledge of the lingual tonsils’ contribution to diseases such as obstructive sleep apnea (OSA). While complications from this procedure are well-studied in the pediatric population, few studies have investigated the morbidity of this procedure in adults. 

Lingual tonsils are a component of Waldeyer’s ring in conjunction with the pharyngeal tonsils (adenoids), tubal tonsils, and palatine tonsils [1]. Lingual tonsils are located at the base of the tongue and are anatomically bound by the epiglottis posteriorly, the circumvallate papillae anteriorly, and the tonsillar pillars laterally [2]. The arterial blood supply is primarily from the lingual branch of the external carotid arteries, while the venous drainage is to the lingual veins of the internal jugular vein. The lymphatic drainage is to the superior deep cervical or jugular lymph nodes.  The relatively larger presence of Waldeyer’s ring lymphoid tissue in childhood is attributed to the developing immune system. In adulthood, immune activity tends to be reduced, and typically these lymphoid tissues gradually regress [3].  However, lingual tonsils may reach functional activity later than other components of Waldeyer’s ring and thus may persist to varying degrees in older ages [4]. Additionally, it is thought that chronic inflammatory processes may contribute to lingual tonsil hypertrophy (LTH). Allergic rhinitis, chronic infections, alcohol, tobacco, and gastroesophageal reflux have all been associated with LTH [5]. Compensatory hyperplasia of lingual tonsils after removal of other components of Waldeyer’s ring has also been theorized [6]. 

LTH has been commonly cited as a contributing factor in patients with OSA, and consequently, OSA is the most common indication for undergoing LT [7]. In a review of 602 LT cases by Merna et al., 58.2% of procedures were performed for OSA, 18.5% for LTH or infection, and 15.2% for malignancy [7]. In contrast to other components of Waldeyer’s ring, which form discrete masses of lymphoid tissue, the lingual tonsils form a non-discrete mass that has traditionally made removal technically challenging [8]. The development of new surgical technology and techniques has increased the popularity of this procedure in recent years. Microdebrider, coblation, sharp dissection, laser, suction diathermy, and trans-oral robotic surgery (TORS) are all commonly used, with no method being accepted as clearly superior in the current literature. While pediatric outcomes for lingual tonsillectomies have been studied extensively, data for adult populations has been limited to small, institution-specific reviews with a focus on the specific surgical technique used [2,8-12]. Of these reports, the most common postoperative complication was bleeding, occurring in up to 9.3% of patients as found in one study [10]. Another study reported additional complications, including persistent gagging sensation, weight loss secondary to poor oral intake, lingual edema, hypoglossal nerve weakness, difficulty swallowing, transient change in taste, and severe respiratory difficulty [13]. 

This study aimed to evaluate postoperative morbidity, mortality, and predictors of prolonged hospital stay in adult patients undergoing lingual tonsillectomy. Given the increasing frequency of this procedure and limited data on adult outcomes, our findings provide insight into short-term complications and patient-specific risk factors using a large, multi-institutional surgical database.

## Materials and methods

Database description 

This study employed the American College of Surgeons National Surgical Quality Improvement Program (ACS-NSQIP) database, a validated, multi-institutional registry designed to measure and improve the quality of surgical care through prospective, risk-adjusted data collection. The program captures data on more than 150 preoperative, intraoperative, and 30-day postoperative variables from over 700 participating hospitals across the United States. Patients under the age of 18 are excluded, and data were collected by trained surgical clinical reviewers using standardized definitions to ensure accuracy and comparability. Cases are selected using a systematic sampling process known as the “eight-day cycle,” in which sites abstract approximately 35 consecutive cases from operative logs every eight days to minimize selection bias. The 2019 Participant Use Data File includes more than one million adult surgical cases and 274 standardized variables [14].

Study design and subjects

This retrospective study protocol was reviewed and approved by the Institutional Review Board for the State University of New York (SUNY) at Buffalo. Using the ACS-NSQIP database, we collected data from 2014 through 2022 on adult patients aged 18 years and older who underwent lingual tonsillectomy (LT). Patients were identified using the Current Procedural Terminology (CPT®) code 42870 as the primary procedure, defined as “excision or destruction of lingual tonsil by any method” [15]. Concurrent procedures were included but analyzed descriptively. Clinical data extracted included patient demographics, medical comorbidities, malignancy, length of stay, readmission, reoperation, and postoperative complications; the American Society of Anesthesiologists (ASA) classification system was also included. The ACS-NSQIP dataset contains a variable for “bleeding disorder,” referring to congenital or acquired coagulopathies; however, it does not capture medication-specific variables such as preoperative anticoagulant or antiplatelet use, which could not be assessed in this analysis. Data were obtained in accordance with the ACS-NSQIP data request process, and definitions for each variable are available in the ACS-NSQIP Participant Use Data File (PUF) [14].

Measures of outcome assessments 

Variables examined include age, gender, body mass index (BMI), and comorbidities. Comorbidities examined included: diabetes, history of smoking, chronic obstructive pulmonary disease (COPD), and hypertension treated with medications. Outcomes of interest included postoperative complications, length of stay, readmission, and reoperation.  

Statistical analyses 

Quantitative data analyses were conducted using SPSS 29 (Version 29.0.0.0; IBM Inc., Armonk, US) and XLSTAT (Version 2021.2.2; Addinsoft, Paris, France). Descriptive statistics were used to summarize demographic data. Data for sex, ethnicity, inpatient versus outpatient status, complications, and comorbidities were reported as raw numbers with percentages. Data on age and BMI were presented as averages with associated confidence intervals. Additionally, regression analysis was done to compare several preoperative comorbidities, including diabetes, history of smoking, COPD, use of anti-hypertensive medications, and congestive heart failure to total hospital length of stay. Comorbidities that were absent for all subjects were excluded from the analysis (ascites, renal failure, and dialysis). 

## Results

Demographics 

A total of 232 patients who underwent LT were identified. Detailed demographic information is shown in Table 1. Cases submitted per year were as follows: in 2022, 25 cases, in 2021, 20 cases, in 2020, 25 cases, in 2019, 22 cases, in 2018, 33 cases, in 2017, 27 cases, in 2016, 36 cases, in 2015, 22 cases, and in 2014, 22 cases. The mean age at the time of surgery was 51.6 (SD=15.1, 95% CI: 49.6-53.5) years and ranged from 18 to 87 years. Mean BMI was 31.0 (SD=6.8, 95% CI: 30.1-31.8), with a range of 12.5 to 52.6. 

**Table 1:** Overall demographics of the patient population Demographics of the patient population, including sex, race, ethnicity, age, BMI, and status as an inpatient or outpatient.

Demographics	Total, n (%) or mean (95% CI)
Sex	
Male	103 (44.4)
Female	129 (55.6)
Race	
White	186 (80.2)
Black or African American	20 (8.6)
Unknown	19 (8.2)
Asian	3 (1.3)
American Indian or Alaskan	2 (0.9)
Native Hawaiian or Pacific Islander	1 (0.4)
Ethnicity	
Hispanic	13 (5.6)
Not Hispanic	200 (86.2)
Unknown	19 (8.2)
Mean age (years)	51.6 (95% CI: 49.6-53.5)
Mean BMI	31.0 (95% CI: 30.1-31.8)
Status	
Inpatient	61 (26.3)
Outpatient	171 (73.7)

Medical comorbidities

Comorbidities are listed in Table 2. The most common comorbidity was hypertension treated with medication, present in 76 patients (32.8%). One hundred eighty-four patients (79.3%) had surgery for benign disease, 46 (19.8%) for malignant disease, and two (0.9%) for neoplasms of uncertain behavior. 

**Table 2:** Comorbidities Listed are the most common comorbidities in patients undergoing lingual tonsillectomy. The most common comorbidities were hypertension, current smoking, and diabetes. COPD - chronic obstructive pulmonary disease; CHF - congestive heart failure

Comorbidity	Total, n (%)
Hypertension	76 (32.8)
Current smoker within one year	48 (20.7)
Diabetes	30 (12.9)
Type I	13 (5.6)
Type II	17 (7.3)
Disseminated cancer	10 (4.3)
History of severe COPD	8 (3.4)
Steroid use for chronic condition	8 (3.4)
Bleeding disorders	5 (2.2)
Systemic sepsis	2 (0.9)
History of CHF	1 (0.4)

Surgical considerations 

The mean total operation time was 71.3 (SD=54.9, 95% CI: 64.2-78.4) minutes. A total of 103 (44.4%) patients had at least one concurrent or other procedure performed, ranging from laryngoscopy and biopsy to radical neck dissection (Table 3). ASA classification ranged from one to four, with 18 (7.8%) in class 1 (no disturbance), 103 (44.4%) in class 2 (mild disturbance), 104 (44.8%) in class 3 (severe disturbance), and 7 (3.0%) in class 4 (life-threatening).

**Table 3:** Common concurrent procedures In this patient population, concurrent procedures were often performed with lingual tonsillectomy. The most common procedures were scopes of the upper respiratory and gastrointestinal tracts.

Clinical variable	Total, n (%)
Laryngoscopy with intervention	35 (15.1)
Laryngoscopy without intervention	24 (10.3)
Esophagoscopy	12 (5.2)
Nasal surgery	12 (5.2)
Tonsillectomy	9 (3.9)
Neck dissection	6 (2.6)
Lymph node biopsy	3 (1.3)
Glossectomy	2 (0.9)

Length of stay, readmission, and reoperation 

Total mean length of stay was one day (SD=2.0, 95% CI: 0.7-1.2), and the total median length of stay was one day (range 0-19). Among inpatient cases, the mean length of stay was 2.4 days (SD=3.4). For outpatient cases, the mean length of stay was 0.5 days (SD=0.7).  Linear regression relating length of stay to patient characteristics and comorbidities revealed that diabetes was significantly associated with increased length of stay (Beta=1.3, 95% CI: 0.4-2.1, p=0.004; Table 4). Three cases (1.3%) returned to the operating room, one for a postoperative hemorrhage, one for esophageal obstruction, and one for an undocumented reason. Finally, there were three (1.3%) procedure-related readmissions, one for pneumonia, one for throat pain, and one for a hemorrhage 12 days post-procedure.

**Table 4:** Total length of stay related to clinical characteristics of patients Of the clinical characteristics and comorbidities within this patient population, only diabetes was significantly associated with increased length of stay. All other characteristics had insignificant associations, either for increased or decreased length of stay. COPD - chronic obstructive pulmonary disease; CHF - congestive heart failure

Clinical variable	Increase in length of stay	Lower 95% CI	Upper 95% CI	p-value
Hypertension	-0.076	-0.683	0.531	0.805
Smoker	0.374	-0.302	1.050	0.277
Diabetes	1.252	0.407	2.098	0.004
Disseminated cancer	-0.418	-1.697	0.861	0.520
COPD	-0.245	-1.743	1.253	0.747
Steroid use	-0.364	-1.773	1.045	0.611
Bleeding disorder	0.078	-1.934	2.089	0.939
Systemic sepsis	2.255	-0.590	5.100	0.120
CHF	0.842	-3.597	5.280	0.709

Complications 

Complications are listed in Table 5. The most common complications were pneumonia (n=3, 1.3%) and urinary tract infections (n=3, 1.3%). There were two cases of postoperative hemorrhage within 30 days in this group, but no bleeds required transfusion. 

**Table 5:** Postoperative complications by surgical indication Of the 232 patients who underwent lingual tonsillectomy, few had postoperative complications.

Complication	Total, n (%)	Airway obstruction, n (%)	Cancer, n (%)
Pneumonia	3 (1.3)	2 (0.9)	0
Urinary tract infection	3 (1.3)	2 (0.9)	0
Unplanned intubation	2 (0.9)	0	0
On ventilator (>48 hours)	2 (0.9)	0	0
Cardiac arrest (requiring CPR)	1 (0.4)	0	0
Septic shock	1 (0.6)	0	0
Superficial surgical site infection	0	0	0
Bleeding requiring transfusion	0	0	0

## Discussion

Rates of morbidity and mortality 

The limited morbidity following adult LT observed in this study contrasts with past analyses reporting significant morbidity associated with this procedure. For example, the study performed by Merna et al. reported postoperative hemorrhage to be the most common cause of readmission, occurring in 1.9% of all cases [7]. Earlier retrospective studies have had similar findings, with a postoperative hemorrhage rate as high as 9.3% [10]. In our study, two of 232 patients (0.9%) had postoperative hemorrhage. Neither of these hemorrhages was reported to require transfusion. There are several reasons that may explain this discrepancy. First, 73.7% of our study’s patients were treated as outpatients. While the severity of each patient’s clinical disease prior to surgery was not objectively identified in our data, the largely outpatient makeup may suggest that operating surgeons identified the overall health of patients as low risk. However, the ASA classification system grouped most patients in this study in class 2 (n=103, 44.4%) or class 3 (n=104, 44.8%). The large number of patients in class 2 and class 3 could suggest that this cohort was at higher risk for postoperative anesthesia complications. 

Much of the current literature on LT focuses on the pediatric population, and few large reviews account for efficacy and morbidity in adults. Those adult studies that do exist are mainly limited to institution-specific reviews of outcomes for a specific surgical technique and/or for adults of a specific characteristic, such as ethnicity or clinical indication (i.e., OSA) [2,10,13,16,17]. To our knowledge, only the study by Merna et al. investigated postoperative complications amongst the adult population undergoing LT for any reason [7]. 

Rates of pneumonia and other infections, sepsis, and mortality are rarely reported in existing literature. Barakate and Havas noted minor postoperative infection in three of 28 cases; however, the infections themselves were unspecified [8]. Even in pediatric populations, a meta-analysis by Kang et al. reported only one patient out of 73 who underwent LT and developed postoperative pneumonia; this patient had Noonan syndrome and was thus medically complex [18,19]. Though not significantly associated with any patient characteristic, this study’s postoperative pneumonia rate of 1.3% represents three patients who underwent LT as inpatients and remained in the hospital for at least one day following LT. 

Wahba et al. emphasized the importance of understanding polysomnographic outcomes following LT, particularly for OSA patients [20]. Their findings on the variations in polysomnographic indices, including the apnea-hypopnea index (AHI), can enrich our understanding of LT's efficacy in treating OSA. In our study, the improved outcomes with a lower complication rate may better reflect preoperative patient selection and advancements in surgical techniques. Baptista et al. highlighted the significance of careful patient selection and preoperative considerations for trans-oral robotic surgery (TORS) as part of multilevel surgery in OSA [21]. This is relevant to our findings, in which the relatively low complication rates could be attributed to meticulous preoperative planning and patient stratification, optimizing surgical outcomes. 

There was one mortality in this group, an older diabetic patient with cancer, and this patient accounts for many of the complications noted: pneumonia and septic shock two days postoperatively, followed by a failure to wean off the ventilator, reintubation, and ultimately cardiac arrest requiring cardiopulmonary resuscitation. The combination of this patient’s older age, comorbidities, and extended operation time with a concurrent procedure - a modified radical neck dissection-increased the risk of complications. While each patient characteristic in our data was individually observed to bear no statistical significance on outcomes, it is not yet known if any specific combination of several characteristics is associated with increased morbidity or mortality.  

Length of stay, readmission, and reoperation 

Our length of hospital stay mean was consistent with rates previously reported in the literature [8]. There were three related readmissions: one for pneumonia, one for throat pain (ICD-10 R07.0), and one for hemorrhage (ICD-10 R58). The pneumonia case was readmitted on the third postoperative day, the throat pain case was readmitted on the sixth postoperative day, and the hemorrhage was readmitted on postoperative day 12. Merna et al. reported an 8.2% readmission rate, with the most common diagnoses being bleeding, dysphagia, fever, nausea, vomiting, diarrhea, acute pain, and airway obstruction [7]. The pneumonia readmission case is possibly a complication related to surgery, as this patient had a preoperative diagnosis of OSA and was a current smoker. Few studies on the complications of LT have included pneumonia as a complication; however, Barakate et al. reported “minor infection” in 1% of patients undergoing LT, but did not specify the nature of the infection [8]. Our study’s findings on lingual tonsillectomy (LT) complications and patient outcomes also align closely with the literature on TORS for OSA [21]. The mean length of stay in our cohort was consistent with previous reports, such as those by Baptista et al., who emphasized the significance of careful patient selection to optimize length of stay and minimize complications [21]. The readmission and reoperation rates in our study, particularly for complications like postoperative hemorrhage and pneumonia, are comparable to the 5.3% major complication rate reported by Baptista et al [21]. Additionally, the presence of concurrent procedures in nearly half of our patients reflects the complexity of LT surgeries, paralleling the multilevel surgical approach often necessary in TORS for OSA patients, as highlighted by Baptista et al. This underscores the necessity of comprehensive surgical planning and individualized risk assessments to improve postoperative outcomes in both LT with and without TORS. 

Three cases returned to the operating room: one due to a postoperative hemorrhage (ICD-10 J95.830), one due to esophageal obstruction (ICD-10 K22.2), and one for an undocumented reason. The postoperative hemorrhage patient was returned to the operating room on postoperative day 20, and the patient with esophageal obstruction returned to the operating room on postoperative day 11 for esophagoscopy (CPT 43196). Esophageal obstruction is not a commonly reported complication of lingual tonsillectomy. However, Cho et al. reported that seven patients (44%) experienced difficulty swallowing for at least one month following surgery [22]. Similarly, Merna et al. found that 1.7% of patients reported dysphagia from postoperative day 1 to the first postoperative follow-up visit [7]. The undocumented case returned to the operating room on postoperative day 13. The undocumented case had a total length of stay of 19 days, had an unexpected reintubation on the fifth postoperative day, failed to wean off the ventilator, and had a postoperative diagnosis of sepsis from an unknown organism (ICD-10 A41.9). Given the available information, we cannot speculate on the purpose of returning to the operating room. 

Although our results report lower morbidity than previous studies, this raises questions about which specific risks influence morbidity in LT. In prior analyses of LT in OSA patients, for example, patients were compared pre- and postoperatively based on LTH severity, grade of tongue base narrowing, and/or AHI [11,22]. Friedman et al. proposed using CT scans to determine lingual tonsil tissue thickness as a method of stratifying the severity of disease in patients with OSA or laryngopharyngeal reflux [23]. Whether or not a similar classification system can be extrapolated to predict risk of postoperative complications is unknown, but it may be worth investigating in future studies on LT for patients with various clinical indications. 

Limitations 

Retrospective analyses using large databases are informative but have inherent limitations. Although data extractors at NSQIP-participating hospitals are highly trained, coding errors cannot be completely eliminated. Additionally, ACS-NSQIP collects general surgical data rather than procedure-specific details; information on surgical technique (e.g., transoral robotic surgery, coblation, cold dissection), operative indication (e.g., obstructive sleep apnea vs malignancy), and pharmacologic factors such as anticoagulant or antiplatelet use are not captured, which may represent unmeasured confounders. The database primarily includes large tertiary centers that can afford participation fees ranging from $10,000 to $29,000 [24], which may introduce selection bias. Finally, only 30-day postoperative outcomes are available, so delayed complications such as persistent dysphagia or taste alteration could not be evaluated. Despite these limitations, ACS-NSQIP remains a valuable resource for assessing rare surgical procedures in a large, multicenter setting.

## Conclusions

Within the constraints of a retrospective database analysis, lingual tonsillectomy in adults was associated with low short-term morbidity and mortality. No significant associations were identified between patient comorbidities and postoperative complications, prolonged hospitalization, or reoperation. These findings suggest, but do not establish causation, that improved patient selection and advancements in surgical technique may contribute to reduced complication rates. Future studies incorporating surgical technique, anticoagulant use, and long-term outcomes are warranted to further characterize the safety profile of lingual tonsillectomy.
